# Mapping dynamic QTL for plant height in triticale

**DOI:** 10.1186/1471-2156-15-59

**Published:** 2014-05-19

**Authors:** Tobias Würschum, Wenxin Liu, Lucas Busemeyer, Matthew R Tucker, Jochen C Reif, Elmar A Weissmann, Volker Hahn, Arno Ruckelshausen, Hans Peter Maurer

**Affiliations:** 1 State Plant Breeding Institute, University of Hohenheim, Stuttgart 70599, Germany; 2 Crop Genetics and Breeding Department, China Agricultural University, Beijing 100193, China; 3 Competence Centre of Applied Agricultural Engineering COALA, University of Applied Sciences Osnabrück, Osnabrück 49076, Germany; 4 ARC Centre of Excellence for Plant Cell Walls, University of Adelaide, Waite Campus, Urrbrae, SA 5064, Australia; 5 Leibniz Institute of Plant Genetics and Crop Plant Research (IPK), Gatersleben 06466, Germany; 6 Saatzucht Dr. Hege GbR Domäne Hohebuch, Waldenburg 74638, Germany

**Keywords:** Plant height, QTL mapping, Dynamic QTL, BreedVision, Precision phenotyping

## Abstract

**Background:**

Plant height is a prime example of a dynamic trait that changes constantly throughout adult development. In this study we utilised a large triticale mapping population, comprising 647 doubled haploid lines derived from 4 families, to phenotype for plant height by a precision phenotyping platform at multiple time points.

**Results:**

Using multiple-line cross QTL mapping we identified main effect and epistatic QTL for plant height for each of the time points. Interestingly, some QTL were detected at all time points whereas others were specific to particular developmental stages. Furthermore, the contribution of the QTL to the genotypic variance of plant height also varied with time as exemplified by a major QTL identified on chromosome 6A.

**Conclusions:**

Taken together, our results in the small grain cereal triticale reveal the importance of considering temporal genetic patterns in the regulation of complex traits such as plant height.

## Background

Plant height in small grain cereals is an important agronomic trait affecting crop performance, particularly lodging and consequently grain yield and grain quality. The reduction of crop height has therefore been an important breeding goal for many decades [1]. The identification of variants that reduce height without adversely affecting yield potential is still an important breeding target. Today, crop height in small grain cereals can be strongly modified by plant breeders through major dwarfing or semi-dwarfing genes. The introduction of the so-called *Reduced height* (*Rht*) genes into bread wheat was a key component of the ‘green revolution’. Alleles of *Rht-B1* and *Rht-D1* are nowadays the major sources of semi-dwarfism in wheat and actually increase grain yield in most environments [2,3]. The *Rht-1* loci encode DELLA proteins that integrate hormonal (gibberellin) and environmental signals to affect plant growth [4]. *Rht-1* is encoded by three homoeoloci located on group 4 chromosomes of the A, B and D genome of wheat [3]. Two further important *Rht* genes, *Rht7* and *Rht8*, were found to be located on chromosomes 2A and 2D, respectively, and may represent a homoeologous series [5]. In rye, the major dwarfing gene *Ddw1* is located on chromosome 5R and may represent a homeolog of *Rht12*[5-7]. *Ddw1* has recently been shown to segregate in elite triticale germplasm and to exhibit a strong effect on plant height [8,9]. In addition, the photoperiod insensitive alleles of the major photoperiod regulator *Ppd-1*, located on group 2 chromosomes, can also have pleiotropic effects on plant height [10].

The genetic control underlying plant height is usually studied by assessing the trait once the plants have reached their final height. Plant height, however, is a dynamic trait [11] that shows strong phenotypic changes during the adult plants’ development. Yan et al. [12] performed QTL mapping in a rice doubled haploid population phenotyped for plant height at multiple time points. This analysis revealed some QTL that were detected at all examined time points but also many QTL that could only be identified at one or some time points. Further support for the temporal changes of the genetic control underlying dynamic traits came from a recent study on biomass in triticale [13]. This study revealed that the entire genetic architecture of biomass accumulation is under temporal dynamic control.

In this study we employed triticale (×*Triticosecale* Wittmack; 2n = 6x = 42; AABBRR) as a model species for small grain cereals and assessed plant height at three different time points by the precision phenotyping platform ‘BreedVision’ [14]. A large doubled haploid population with 647 individuals derived from 4 families formed the basis for multiple-line cross QTL mapping [15] to identify plant height QTL at all three time points. In particular our objectives were to identify main and epistatic QTL, assess the contribution of these QTL to the genotypic variance of plant height, and to unravel the temporal genetic patterns underlying the phenotypic development of plant height.

## Results

**Figure 1:**
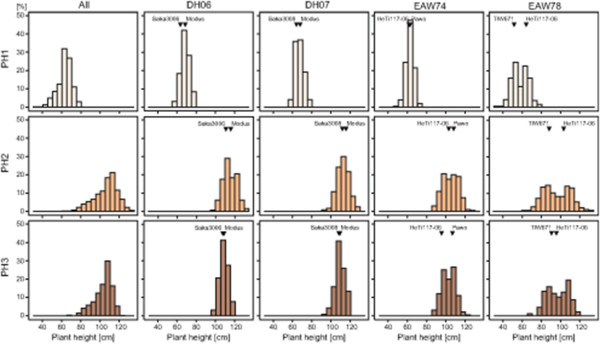
**Phenotypic development of plant height.** Histograms of the phenotypic values for plant height at three developmental stages (PH1-PH3) for the entire population (All) and for each of the four families (DH06, DH07, EAW74, EAW78). The arrowheads indicate the phenotypic values of the parents.

In the mapping population with 647 triticale DHs derived from 4 families we observed significant (*P* < 0.01) genotypic variances $\left(\sigma_{G}^{2}\right)$ and genotype-by-environment interaction variances $\left(\sigma_{G\times E}^{2}\right)$ for predicted plant height at all three developmental stages (PH1 – PH3) (Table 1). The heritabilities were high and ranged from 0.91 for PH3 to 0.96 for PH2. The coefficient of determination (*R*^*2*^) values between plant height at the three time points ranged from 0.72 for PH1-PH3 to 0.88 between PH2 and PH3 (Additional file 1: Figure S1). The parents of the four families differed in their phenotypic values to varying degrees (Figure 1) and for PH1 and PH2 family EAW78 showed the largest difference between the parental lines. Orthogonal contrasts of the means of the families and their respective parents were not significant for any of the three time points. The trait distributions approximately followed a normal distribution except for family EAW78 for which the distribution at all three time points showed a slight bimodal tendency. For all three plant height measurements and all families we observed DH lines that transgressed their respective parents.

**Table 1:** Summary statistics for plant height (cm) at the three developmental stages (PH1-PH3)

	**PH1**	**PH2**	**PH3**
Min	30.05	60.40	65.72
Mean	62.79	105.70	102.90
Max	79.98	131.00	120.50
$\sigma_{G}^{2}$	50.58**	144.35**	76.50**
$\sigma_{G\times E}^{2}$	12.05**	10.26**	13.81**
$\sigma_{e}^{2}$	6.22	15.91	25.58
*h*^2^	0.93	0.96	0.91

Genotypic variance $\left(\sigma_{G}^{2}\right)$, genotype-by-environment interaction variance $\left(\sigma_{G\times E}^{2}\right)$, error variance $\sigma_{e}^{2}$ and heritability (*h*^*2*^). **significant at *P* < 0.01.

**Figure 2:**
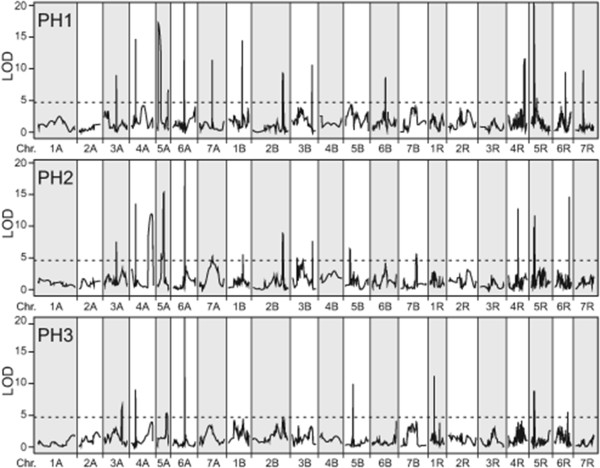
**QTL for plant height at three developmental stages (PH1-PH3).** The dashed horizontal line indicates the significance threshold.

**Figure 3:**
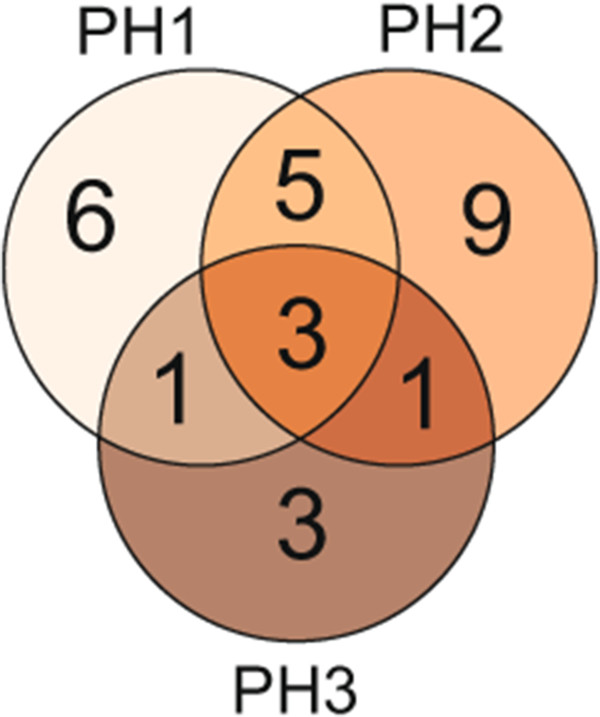
**Venn diagram.** Venn diagram for QTL detected for plant height at three developmental stages (PH1-PH3).

Employing multiple-line cross QTL mapping we identified 15 QTL for PH1, 18 for PH2 and 8 for PH3 (Table 2, Figure 2). Interestingly, we observed three QTL for plant height that were identified at all three time points, some that were identified for two time points but also QTL that were specific for either PH1, PH2 or PH3 (Figure 3). Together the detected QTL explained 77.7, 78.3 and 73.1% of the genotypic variance of PH1, PH2 and PH3, respectively (Table 2). The proportion of genotypic variance explained by single QTL ranged from 1.4 to 44.0% for PH1, from 0.4 to 41.7% for PH2 and from 1.5 to 48.6% for PH3 (Additional file 1: Table S1). The major QTL for all three time points was the QTL on chromosome 5R. Another major QTL explaining more than 5% of the genotypic variance at PH2 and PH3 was identified on chromosome 6A. We used fivefold cross-validation to obtain asymptotically unbiased estimates of the proportion of genotypic variance explained by the detected QTL. Cross-validated, the QTL still explained 55.3, 58.2 and 56.0% of the genotypic variance of PH1, PH2 and PH3, respectively (Table 2). The QTL frequency distributions revealed that most QTL detected with the full data set could be identified in a high number of the runs whereas some were only detected in few runs (Additional file 1: Figure S2).

**Table 2:** Results of QTL mapping at three developmental stages (PH1-PH3) and fivefold cross-validation

	**PH1**	**PH2**	**PH3**
QTL_DS_	15	18	8
*p*_G-DS_	77.7	78.3	73.1
QTL_ES_	14.4	13.1	7.8
*p*_G-ES_	77.2	76.1	70.4
*p*_G-TS_	55.3	58.2	56.0
Relative bias	28.4	23.5	20.5

Number of detected QTL (QTL_DS_), proportion of genotypic variance (%) explained by the detected QTL across all families in the data set (*p*_G-DS_), number of QTL (QTL_ES_) and proportion of genotypic variance averaged over estimation sets (*p*_G-ES_) and averaged over test sets (*p*_G-TS_), and relative bias (%) in the estimation of *p*_G_.

**Figure 4:**
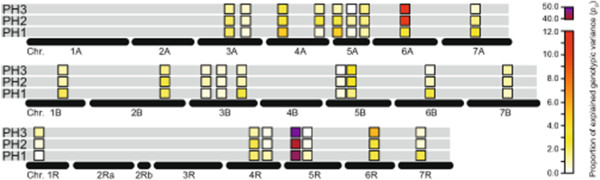
**Temporal contribution of QTL.** Temporal development of the contribution of the QTL detected for any of the three time points (PH1-PH3) to the proportion of explained genotypic variance for plant height in the entire population.

We next assessed temporal changes in QTL contribution to the genotypic variance (*p*_*G*_) of plant height for all QTL detected at any of the three time points (Figure 4). This analysis revealed that for some QTL the *p*_*G*_ remained on a rather constant level while for others it substantially changed between the three time points.

**Figure 5:**
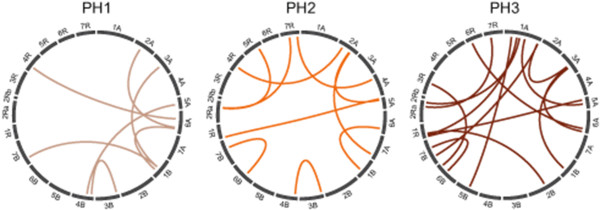
**Epistatic QTL for plant height at three developmental stages (PH1-PH3).** Following the approach suggested by Holland et al. [37] the significance threshold was set at *P* < 5.3e-5.

The full 2-dimensional epistasis scan identified epistatic QTL for plant height for all three time points (Figure 5). Some of these were specific for one time point whereas others were identified at more than one time point. The contribution of these epistatic QTL to the genotypic variance was rather small, ranging between 0.0 and 1.4%.

## Discussion

Many traits of agronomic or biological importance undergo dynamic phenotypic changes during development. Plant height in cereals is a prime example for such a dynamic trait as it changes constantly throughout most of the plants’ adult life. Nevertheless, studies on the genetic control of plant height have so far mainly focussed on the final height, thus neglecting the trait’s developmental dynamics. In this study we therefore investigated plant height at three different time points to unravel the genetic dynamics underlying plant height development.

### Phenotypic development of plant height

A prerequisite for dynamic QTL mapping is the assessment of the trait at multiple time points. In this study, we used the recently described ‘BreedVision’ precision phenotyping platform [14] to assess plant height at three different time points, approximately corresponding to the stages when awns become visible, late flowering and very early dough development [16]. Based on an elaborate calibration experiment and employing triticale as model species, this precision phenotyping platform has been shown to accurately predict plant height [14]. In this study, we observed high heritabilities above 0.9 for predicted plant height at all three time points, based on a large mapping population evaluated at 4 environments. These high heritabilities in combination with the high prediction accuracy demonstrate the great potential of precision phenotyping, especially for a temporal assessment of traits as required to dissect their underlying genetic dynamics.

The *R*^*2*^ values between plant height at the three time points were highest for the contiguous time points and decreased slightly with increasing time between measurements (Additional file 1: Figure S1). This is comparable to the temporal development of another dynamic trait, biomass yield, in triticale [13]. These results indicate that although the measurements at the different time points are not independent, there is variation that is unaccounted for. These differences between genotypes in phenotypic development thus indicate differences in the underlying genetics. The trait distributions approximately followed a normal distribution except for family EAW78 (Figure 1). This family has recently been shown to segregate for a major plant height QTL on chromosome 5R [8] which corresponds to the rye dwarfing gene *Ddw1*[9]. This major QTL explained 40 - 50% of the genotypic variance at all three time points and is thus the most likely reason for the observed bimodal tendency in this family. Taken together, these results illustrate the phenotypic plasticity of plant height during development which underlines the need for a temporal assessment of dynamic traits.

### The genetic architecture of plant height

The QTL with the highest contribution to the genotypic variance were identified on chromosomes 5R and 6A (Additional file 1: Table S1) and the QTL on chromosome 5R segregating in family EAW78 has recently been identified as *Ddw1*[9]. This illustrates that the rye derived *Ddw1* also exhibits a strong effect in triticale in the presence of the wheat A and B genomes. The QTL on chromosome 6A does not appear to represent one of the known major *Rht* loci and may be a novel QTL. No QTL was detected on chromosome 4B where *Rht-B1* is located in wheat [3].

The QTL frequency distributions further supported the majority of the QTL as they were identified in a high number of runs. The cross-validated proportion of genotypic variance explained by the detected QTL amounted to a considerable 55.3 to 58.2% (Table 2). This can to some extent be explained by the major QTL on chromosome 5R but must also be attributed to other QTL spread over the entire genome. Our results thus indicate that plant height in triticale is a complex trait controlled by some major or medium effect QTL in addition to many small effect QTL.

Epistasis refers to interactions between the alleles at two or more genetic loci in the genome [17]. Epistatic QTL contribute to the genetic architecture of many complex traits and have recently been reported for different crops including wheat, maize, and rapeseed [18-22]. The orthogonal contrasts between family means and the means of the respective parents can indicate the presence of epistasis. Non-significant orthogonal contrasts as observed here, however, do not exclude the presence of epistasis and the full 2-dimensional epistasis scans did indeed detect epistatic QTL for each of the three time points. The contribution of these epistatic interactions to the genotypic variance was rather small. It must be noted however that the power to detect epistatic QTL more strongly depends on population size than the power to detect main effect QTL and consequently many epistatic QTL may have remained undetected. Thus, while their individual contribution to the genotypic variance of plant height may be small, their combined contribution may be substantial assuming a higher number of epistatic interactions. Collectively, these results show that both main effect and epistatic QTL contribute to the genetic architecture of plant height in triticale.

### Temporal genetic dynamics underlying plant height development

Dynamic traits are characterized by temporal phenotypic changes suggesting that the genetic control underlying such traits may also vary with time. QTL mapping for plant height in rice at multiple time points revealed many loci that were specific for one or a few of the time points [12]. Employing triticale as model species for small grain cereals, Busemeyer et al. [13] and has recently reported dynamic QTL mapping for biomass and also reported developmental stage specific QTL.

In this study we assessed plant height of triticale at three different time points. Our results corroborate those from rice [12], as we also identified QTL that appear to contribute to the genetic control underlying plant height at all investigated time points, whereas other QTL were only identified at one or two of the time points. This illustrates that QTL underlying plant height are often not static and the trait not controlled by a fixed set of loci throughout crop height development. Rather, the genetic control of plant height undergoes rapid temporal changes. This is also illustrated by the major QTL on chromosome 6A. While this QTL was identified at all three time points, its contribution to the genotypic variance of plant height increased from 3.5% at PH1 to 10.7% at PH2 and finally 11.5% at PH3 (Figure 4). This suggests that the effects of this QTL become more pronounced after flowering of the plants. While at PH1 many QTL with small effect were identified, the least number of QTL was detected at PH3 for which the majority of the genotypic variance was contributed by the two major QTL on chromosomes 5R and 6A. This illustrates that assessing only the final plant height as represented by PH3 is not fully representative for the genetic architecture underlying plant height and would miss a number of QTL active during earlier development.

Interestingly, we found a similar dynamic genetic pattern for the epistatic interactions which also changed with development (Figure 5). Collectively, our results revealed that the entire genetic architecture underlying plant height shows dynamic temporal changes during crop development.

## Conclusions

In this study, we employed a precision phenotyping platform to assess plant height of triticale at three time points. We show that both main effect and epistatic QTL are not static but rather are mainly detected at only one or two of the examined developmental stages. In addition, we observed variable contributions of QTL to the genotypic variance of plant height, as exemplified by the major QTL detected on chromosome 6A. Taken together, our results illustrate the temporal dynamics of the genetic control underlying plant height which emphasizes the need for multiple assessments of such dynamic traits.

## Methods

### Plant material, field trials and phenotypic data

The plant material and the field trials used in this study have been described in Busemeyer et al. [13]. Phenotypic data for plant height were obtained by non-invasive prediction based on the ‘BreedVision’ precision phenotyping platform [14]. Plant height was predicted at the three developmental stages: PH1 = BBCH stage 49 (awns visible), PH2 = BBCH 69 (late flowering), and PH3 = BBCH 81 (very early dough development) [16], in a mapping population consisting of 647 doubled haploid (DH) [23] triticale lines. The DH lines are derived from four families designated DH06 (131), DH07 (120), EAW74 (200), and EAW78 (196) which have been described by Alheit et al. [24]. The DH lines were grown in partially replicated designs [25] including common checks with 960 plots per location, at two locations in two years. Phenotypic data were analyzed by ordinary alpha analysis of variance [25]. Variance components were determined by the restricted maximum likelihood (REML) method assuming a full random model and heritability (*h*^*2*^) on an entry-mean basis was estimated from the variance components as the ratio of genotypic to phenotypic variance [26]. Best linear unbiased estimates (BLUEs) were estimated across environments assuming fixed effects for the genotype. All statistical analyses were performed using ASReml 3.0 [27].

### Multiple-line cross QTL mapping

The DH lines were genotyped with DArT markers and QTL mapping was done based on the integrated consensus linkage map described by Alheit et al. [24]. For QTL mapping, an additive genetic model was chosen and a joint analysis was performed with a model assuming specific QTL effects for every family [28] as described in detail by Steinhoff et al. [29]. In brief, the multiple-line cross QTL mapping model was:

$$Y=\mathrm{JM}+X_{q}B_{q}+\sum_{c\neq q}X_{c}B_{c}+\epsilon$$

where Y was a *N* × 1 column vector of the BLUE values of phenotypic data of *N* progenies coming from *P* families. J was a *N* × *P* matrix whose elements were 1 or 0 according to whether or not individual *i* belonged to family *p* and *M* was a *P* × 1 vector of family specific means. X_q_ (X_c_) a *N* × *P* matrix containing the expected number (ranging from 0 to 2) of allele *k* for each individual in family *p* at QTL *q* (cofactor *c*), and B_q_ (B_c_) was a *P* × 1 vector of the expected allele substitution effects of QTL *q* (cofactor *c*) in family *p*, ϵ was the vector of the residuals.

Cofactor selection was performed using PROC GLMSELECT implemented in the statistical software SAS [30]. The presence of a putative QTL in an interval was tested using a likelihood-ratio test with the statistical software R [31]. LOD-thresholds of 4.7 for PH1, 4.6 for PH2 and 4.7 for PH3 were used corresponding to an experiment-wise type I error of *P* < 0.10, based on 2,000 permutations [32]. Cofactors were excluded within a distance to the marker interval under consideration smaller than 10 cM and the support interval of a QTL was defined as a LOD fall-off of 1.0 expressed as position on the chromosome in centimorgans (cM) [33]. The proportion of genotypic variance explained by the detected QTL was estimated as *R*^*2*^_*adj*_/*h*^*2*^[34]*.* Plant height QTL were declared as overlapping between the three developmental stages if they fell within an arbitrarily defined 10 cM interval surrounding the QTL. Fivefold cross-validation was done as described previously [35,36]. QTL frequency distributions were assessed by a 5 cM sliding window.

The epistasis scan for pairwise interactions was done with the model described above which was extended by the term X_*q’*_B_*q’*_ for the second locus and the interaction term between the two loci *q* and *q’* X_*qq’*_B_*qq’*_. We used an α-level of 0.05 and followed the suggestion of Holland et al. [37] dividing the α-level by the number of possible independent pairwise interactions between chromosome regions, assuming two separate regions per chromosome (*P* < 5.3e-5). The circular plots illustrating the epistatic interactions were created with Circos [38].

## Competing interests

The authors declare that they have no competing interests.

## Authors’ contributions

TW, JCR, EAW, VH, AR, HPM conceived experiments, LB collected data, TW, WL, LB performed analyses, TW, MRT, JCR wrote the manuscript. All authors read and approved the final manuscript.

## Supplementary Material

Additional file 1: Table S1 QTL detected for plant height at three developmental stages (PH1-PH3). Chromosome, position with support interval and proportion of genotypic variance explained by the QTL (pG in %). **Figure S1.** R^2^ values between plant height at three developmental stages (PH1-PH3). **Figure S2.** QTL frequency distributions. Frequency distributions for the QTL detected at three developmental stages (PH1-PH3) derived from fivefold cross-validation. The arrowheads indicate QTL positions of the full data set. Click here for file
