# Supplementary material for: Mapping dynamic QTL for plant height in triticale
Source: BMC Genet. 2014 May 19;15:59. doi: 10.1186/1471-2156-15-59 (PMC4040121; doi:10.1186/1471-2156-15-59)
Supplement: Additional file 1: Table S1 — QTL detected for plant height at three developmental stages (PH1-PH3). Chromosome, position with support interval and proportion of genotypic variance explained by the QTL (pG in %). Figure S1. R2 values between plant height at three developmental stages (PH1-PH3). Figure S2. QTL frequency distributions. Frequency distributions for the QTL detected at three developmental stages (PH1-PH3) derived from fivefold cross-validation. The arrowheads indicate QTL positions of the full data set. [file 1471-2156-15-59-S1.pdf]

Mapping dynamic QTL for plant height in triticale

Tobias Würschum[1§]*, Wenxin Liu[2]*, Lucas Busemeyer[3], Matthew R. Tucker[4], Jochen C. Reif[5], Elmar A. Weissmann[6], Volker Hahn[1], Arno Ruckelshausen[3], Hans Peter Maurer[1]

[1] State Plant Breeding Institute, University of Hohenheim, 70599 Stuttgart, Germany

[2] Crop Genetics and Breeding Department, China Agricultural University, 100193 Beijing, China

[3] Competence Centre of Applied Agricultural Engineering COALA, University of Applied Sciences Osnabrück, 49076 Osnabrück, Germany

[4] ARC Centre of Excellence for Plant Cell Walls, University of Adelaide, Waite Campus, Urrbrae SA 5064, Australia

[5] Leibniz Institute of Plant Genetics and Crop Plant Research (IPK), 06466 Gatersleben, Germany

[6] Saatzucht Dr. Hege GbR Domäne Hohebuch, 74638 Waldenburg, Germany

§ Corresponding author; * Joint first authors

Supplementary Information

This file contains the Supplementary Tables and Figures.

Table S1 QTL detected for plant height at three developmental stages (PH1-PH3). Chromosome, position with support interval and proportion of genotypic variance explained by the QTL (p_G in %).

	Chromosome	Position in cM [support interval]	p_G
PH1			
	3A	57.8 [57.8 - 58.7]	1.9
	4A	21.2 [21.0 - 21.5]	4.4
	5A	3.5 [3.3 - 10.1]	4.2
	5A	52.5 [51.6 - 52.9]	1.4
	6A	55.5 [55.3 - 56.4]	3.5
	7A	61.5 [61.1 - 61.7]	3.1
	1B	66.7 [66.0 - 67.3]	2.8
	2B	144.5 [142.8 - 147.8]	2.0
	3B	98.1 [97.4 - 98.1]	2.1
	6B	64.7 [64.1 - 65.2]	1.8
	4R	81.7 [77.0 – 81.7]	1.9
	5R	14.4 [13.8 – 15.1]	44.0
	5R	27.5 [26.9 – 27.8]	1.4
	6R	53.5 [53.2 – 53.7]	1.8
	7R	38.1 [37.8 – 38.1]	2.4
PH2			
	3A	57.8 [57.7 - 58.7]	2.4
	4A	20.9 [20.7 - 21.0]	3.3
	4A	98.7 [90.0 - 104.4]	1.5
	4A	106.3 [81.8 - 106.9]	0.4
	5A	18.9 [18.4 - 33.6]	1.1
	5A	31.8 [28.3 - 32.3]	2.8
	6A	58.2 [57.9 - 58.5]	10.7
	7A	61.5 [55.5 - 68.0]	1.0
	1B	70.4 [70.0 - 70.8]	1.0
	2B	144.5 [142.8 - 147.8]	1.8
	3B	24.6 [24.1 – 25.5]	1.1
	3B	53.1 [46.9 – 55.3]	0.5
	3B	100.2 [99.7 – 100.6]	1.2
	5B	21.2 [20.8 – 24.8]	2.5
	7B	78.5 [76.5 – 81.2]	1.2
	4R	48.5 [48.2 – 48.6]	3.3
	5R	17.0 [17.0 – 18.0]	41.7
	6R	72.7 [72.7 – 72.8]	1.6
PH3			
	3A	87.1 [82.2 - 87.2]	1.7
	4A	20.5 [20.4 - 20.7]	2.4
	5A	43.1 [42.3 - 50.1]	1.5
	6A	58.2 [57.9 - 58.5]	11.5
	5B	37.0 [36.5 - 37.5]	3.5
	1R	20.6 [20.5 - 20.6]	2.8
	5R	15.7 [12.7 - 16.3]	48.6
	6R	64.8 [63.9 - 65.0]	1.5

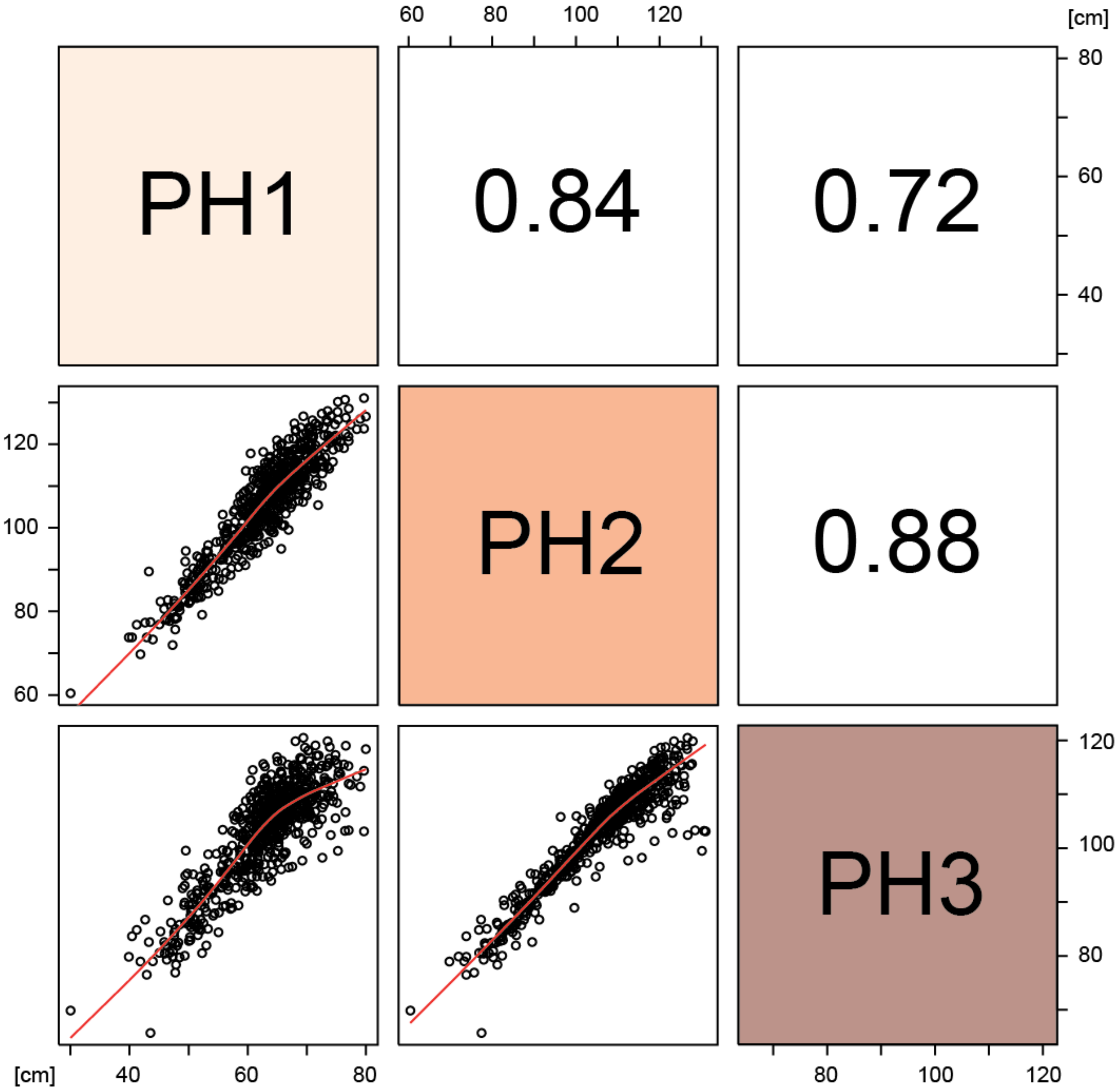

Figure S1 R^2 values between plant height at three developmental stages (PH1-PH3).

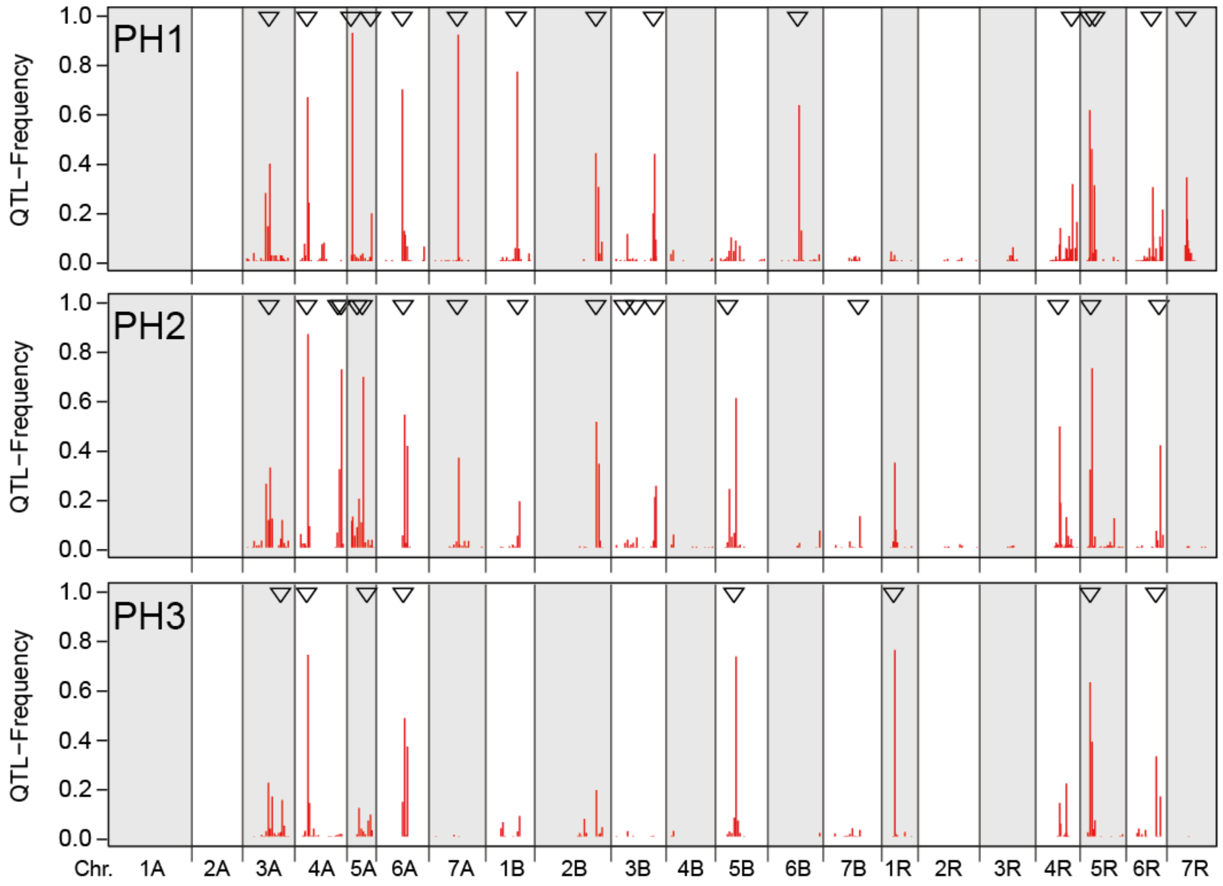

Figure S2 QTL frequency distributions. Frequency distributions for the QTL detected at three developmental stages (PH1-PH3) derived from fivefold cross-validation. The arrowheads indicate QTL positions of the full data set.
